# Antibiotic susceptibility profiles of *Mycoplasma* sp. 1220 strains isolated from geese in Hungary

**DOI:** 10.1186/s12917-016-0799-0

**Published:** 2016-08-19

**Authors:** Dénes Grózner, Zsuzsa Kreizinger, Kinga M. Sulyok, Zsuzsanna Rónai, Veronika Hrivnák, Ibolya Turcsányi, Szilárd Jánosi, Miklós Gyuranecz

**Affiliations:** 1 Institute for Veterinary Medical Research, Centre for Agricultural Research, Hungarian Academy of Sciences, Hungária körút 21, Budapest, 1143 Hungary; 2 Veterinary Diagnostic Directorate, National Food Chain Safety Office, P.O. Box 21581, Budapest, Hungary

**Keywords:** Antibiotic resistance, Duck, Goose, MIC, Microbroth dilution, *Mycoplasma* sp. 1220

## Abstract

**Background:**

*Mycoplasma* sp. 1220 can induce inflammation primarily in the genital and respiratory tracts of waterfowl, leading to serious economic losses. Adequate housing and appropriate antibiotic treatment are promoted in the control of the disease. The aim of the present study was to determine the in vitro susceptibility to thirteen different antibiotics and an antibiotic combination of thirty-eight *M*. sp. 1220 strains isolated from geese and a duck in several parts of Hungary, Central Europe between 2011 and 2015.

**Results:**

High MIC_50_ values were observed in the cases of tilmicosin (>64 μg/ml), oxytetracycline (64 μg/ml), norfloxacin (>10 μg/ml) and difloxacin (10 μg/ml). The examined strains yielded the same MIC_50_ values with spectinomycin, tylosin and florfenicol (8 μg/ml), while enrofloxacin (MIC_50_ 5 μg/ml), doxycycline (MIC_50_ 5 μg/ml), lincomycin (MIC_50_ 4 μg/ml) and lincomycin-spectinomycin (1:2) combination (MIC_50_ 4 μg/ml) inhibited the growth of the bacteria with lower concentrations. Tylvalosin (MIC_50_ 0.5 μg/ml) and two pleuromutilins (tiamulin MIC_50_ 0.625 μg/ml; valnemulin MIC_50_ ≤ 0.039 μg/ml) were found to be the most effective drugs against *M*. sp. 1220. However, strains with elevated MIC values were detected for all applied antibiotics.

**Conclusions:**

Valnemulin, tiamulin and tylvalosin were found to be the most effective antibiotics in the study. Increasing resistance was observed in the cases of several antibiotics. The results highlight the importance of testing *Mycoplasma* species for antibiotic susceptibility before therapy.

## Background

*Mycoplasma* sp. 1220 was first described as a new *Mycoplasma* species by Stipkovits et al. in 1986 [1]. This *Mycoplasma* species causes cloaca and phallus inflammation and testicular atrophy in the ganders [1, 2]. In the infected geese salpingitis and vaginitis are the main symptoms [1, 3]. The egg production activates the pathogen and the flared up mycoplasmosis could induce lethal pathological changes in the embryos [1, 4]. Airsacculitis and peritonitis are also common, and general symptoms such as changes in thirst, decreased food consumption, body weight losses, weakness, nasal discharge, impaired breathing, conjunctivitis, diarrhoea and nervous signs were also described in the affected waterfowl flocks [2, 5–8]. *Mycoplasma* infection of the birds can aggravate diseases caused by other agents and could lead to serious economic losses [3, 6]. Since there is no commercially available vaccine against *M*. sp. 1220, adequate housing and appropriate antibiotic treatment are promoted in the control of the diseases caused by this agent. Prophylactic medication could prevent economic losses if appropriate antibiotics are administered in the early weeks of life and in expected stress periods. Medication of the layers is essential to reduce the vertical transmission of *M.* sp. 1220 [2].

*Mycoplasmas* are resistant to β-lactam antimicrobials because of the lack of cell-wall and the bacteria are also resistant to membrane synthesis inhibitors [2, 9]. Antibiotics such as quinolones, tetracyclines, macrolides and pleuromutilins which induce DNA fragmentation or inhibition at the level of protein synthesis are the drugs of choice for the therapy of mycoplasmosis. Among the macrolides, erythromycin showed high effectiveness against *Mycoplasma* strains which could ferment glucose (e.g. *M.* sp. 1220), while arginine-hydrolysing strains proved to be less susceptible to this compound [2, 10]. *Mycoplasma* infected waterfowl and poultry flocks are usually treated with macrolides, pleuromutilins or with the combination of lincomycin and spectinomycin [3, 11–18].

The aim of this study was to determine the susceptibility of 38 Hungarian *M*. sp. 1220 isolates to thirteen antibiotics and a drug combination using the microbroth dilution method.

## Methods

**Fig. 1:**
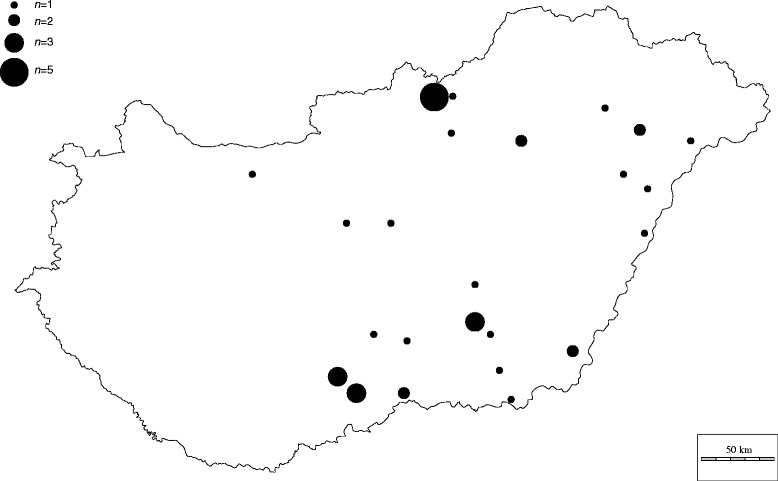
Map of Hungary showing the geographical origin of the *Mycoplasma* sp. 1220 isolates. Size of the circles indicates the number (*n*) of the strains. (The blank map was downloaded from an open source [28])

A total of 38 *M.* sp. 1220 strains isolated from geese and a duck originating from different parts of Hungary were tested in the study (Table 1, Fig. 1). The samples were collected during routine diagnostic examinations or necropsies between 2011 and 2015, thus ethical approval was not required for the study. Phallus lymph, cloaca swabs, tracheal swabs, follicules and lung samples were washed in 2 ml of *Mycoplasma* broth medium (pH 7.8) (ThermoFisher Scientific Inc./Oxoid Inc./, Waltham, MA) supplemented with 0.5 % (w/v) sodium pyruvate, 0.5 % (w/v) glucose and 0.005 % (w/v) phenol red and incubated at 37 °C in a 5 % CO_2_ atmosphere. The cultures were inoculated onto solid *Mycoplasma* media (Thermo Fisher Scientific Inc./Oxoid Inc./) after colour change of the broth, and were incubated at 37 °C and 5 % CO_2_ until visible colonies appeared (1–2 days). Purification of mixed cultures was performed by one-time filter cloning, minimizing the in vitro mutations of the isolates. The QIAamp DNA Mini Kit (Qiagen Inc., Hilden, Germany) was used for DNA extraction according to the manufacturers’ instructions for Gram-negative bacteria. The purity of the cultures was confirmed by a universal *Mycoplasma* PCR system targeting the 16S/23S rRNA intergenic spacer region in *Mycoplasmatales* followed by sequencing on an ABI Prism 3100 automated DNA sequencer (Applied Biosystems, Foster City, CA), sequence analysis and BLAST search [19]. The number of colour changing units (CCU) was calculated by microbroth dilution method, from the lowest dilution showing colour change after one week of incubation [9].

**Table 1:** Background data and MIC values of the isolated *Mycoplasma* sp. 1220 strains

					MIC values (μg/ml)													
					Fluoroquinolones			Aminoglycoside	Lincosamide		Tetracyclines		Macrolides			Pleuromutilines		Phenicol
Sample ID	Sample source	Place	Animal	Date	Enrofloxacin	Norfloxacin	Difloxacin	Spectinomycin	Lincomycin	Lincomycin-spectinomycin (1:2) combination	Oxytetracycline	Doxycycline	Tylosin	Tilmicosin	Tylvalosin	Tiamulin	Valnemulin	Florfenicol
MYCAV 65	Phallus lymph	Rém	goose	2014	5	>10	10	16	4	4	32	5	0.5	0.5	≤0.25	1.25	0.078	8
MYCAV 34	Phallus lymph	Szentes	goose	2011	5	>10	10	8	4	2	64	2.5	1	4	≤0.25	0.625	≤0.039	4
MYCAV 35	Phallus lymph	Rém	goose	2012	5	>10	10	>64	4	4	64	10	1	4	≤0.25	1.25	≤0.039	8
MYCAV 36	Cloaca	Hajdúböszörmény	goose	2012	5	>10	>10	64	4	4	64	>10	1	4	≤0.25	1.25	≤0.039	8
MYCAV 38	Cloaca	Kelebia	goose	2012	2.5	>10	10	8	2	4	4	0.156	≤0.25	≤0.25	≤0.25	0.625	≤0.039	4
MYCAV 44	Cloaca	Nagykamarás	goose	2012	5	>10	10	8	4	4	8	0.312	8	>64	0.5	1.25	≤0.039	8
MYCAV 47	Lung	Tázlár	duck	2012	>10	>10	>10	16	>64	16	>64	5	16	>64	1	2.5	0.312	8
MYCAV 49	Phallus lymph	Tiszavasvári	goose	2013	5	>10	10	16	4	4	64	5	8	>64	0.5	0.625	≤0.039	8
MYCAV 50	Phallus lymph	Cered	goose	2013	>10	>10	>10	16	4	4	>64	5	2	2	≤0.25	0.625	≤0.039	8
MYCAV 51	Phallus lymph	Derekegyház	goose	2013	5	>10	10	32	4	4	>64	10	8	>64	0.5	0.625	≤0.039	8
MYCAV 53	Phallus lymph	Szentes	goose	2013	5	>10	10	16	4	4	>64	10	8	>64	0.5	0.625	≤0.039	8
MYCAV 54	Follicule	Hódmezővásárhely	goose	2013	5	>10	10	8	4	4	>64	5	8	>64	0.5	0.625	≤0.039	8
MYCAV 55	Follicule	Kiskunmajsa	goose	2013	10	>10	10	8	4	4	8	0.312	≤0.25	≤0.25	≤0.25	0.625	≤0.039	4
MYCAV 56	Phallus lymph	Sükösd	goose	2013	1.25	>10	1.25	8	4	4	4	0.312	8	>64	0.5	0.625	≤0.039	4
MYCAV 59	Follicule	Rém	goose	2013	5	>10	10	8	4	4	32	2.5	0.5	≤0.25	≤0.25	1.25	0.078	2
MYCAV 61	Phallus lymph	Tatárszentgyörgy	goose	2013	5	>10	10	16	2	4	2	0.078	≤0.25	≤0.25	≤0.25	0.312	≤0.039	4
MYCAV 63	Trachea	Sükösd	goose	2013	1.25	10	1.25	8	2	2	4	0.312	4	64	≤0.25	0.156	≤0.039	4
MYCAV 66	Phallus lymph	Tiszaföldvár	goose	2014	5	>10	10	16	4	4	>64	>10	≤0.25	≤0.25	≤0.25	0.625	≤0.039	4
MYCAV 67	Phallus lymph	Szentes	goose	2014	5	>10	10	8	>64	16	>64	5	>64	>64	16	2.5	0.078	4
MYCAV 68	Phallus lymph	Érpatak	goose	2014	5	>10	10	8	>64	32	>64	10	>64	>64	16	5	≤0.039	8
MYCAV 69	Phallus lymph	Ludas	goose	2014	5	>10	10	4	4	4	>64	5	8	>64	1	0.625	≤0.039	4
MYCAV 70	Phallus lymph	Cered	goose	2014	>10	>10	>10	16	4	4	>64	>10	16	>64	1	0.625	≤0.039	8
MYCAV 71	Phallus lymph	Sükösd	goose	2014	1.25	>10	1.25	8	2	4	8	0.625	8	>64	0.5	0.625	≤0.039	4
MYCAV 72	Phallus lymph	Nagykamarás	goose	2014	5	>10	10	8	4	4	4	0.312	8	>64	0.5	0.625	≤0.039	4
MYCAV 75	Phallus lymph	Dömsöd	goose	2014	5	>10	10	16	4	4	>64	10	≤0.25	≤0.25	≤0.25	0.625	≤0.039	8
MYCAV 76	Phallus lymph	Tiszabábolna	goose	2014	5	>10	10	32	8	4	64	5	8	>64	0.5	1.25	≤0.039	8
MYCAV 91	Phallus lymph	Hajdúsámson	goose	2011	10	>10	>10	8	8	4	64	2.5	≤0.25	≤0.25	≤0.25	0.625	≤0.039	8
MYCAV 93	Phallus lymph	Bojt	goose	2014	2.5	>10	1.25	8	2	4	8	0.312	≤0.25	≤0.25	≤0.25	0.312	≤0.039	8
MYCAV 94	Cloaca	Tiszabábolna	goose	2012	2.5	>10	5	16	4	4	>64	>10	≤0.25	≤0.25	≤0.25	0.625	≤0.039	8
MYCAV 160	Phallus lymph	Érpatak	goose	2015	>10	>10	>10	16	4	4	>64	10	>64	>64	2	0.625	≤0.039	8
MYCAV 161	Phallus lymph	Szilaspogony	goose	2015	>10	>10	>10	8	4	4	>64	>10	16	>64	0.5	0.625	≤0.039	8
MYCAV 162	Phallus lymph	Encsencs	goose	2015	2.5	>10	10	8	4	4	>64	5	16	>64	0.5	0.625	≤0.039	4
MYCAV 176	Phallus lymph	Cered	goose	2015	10	>10	5	8	4	4	>64	5	64	>64	4	0.625	≤0.039	16
MYCAV 177	Phallus lymph	Cered	goose	2015	>10	>10	10	8	4	4	>64	10	>64	>64	4	0.625	≤0.039	32
MYCAV 178	Follicule	Cered	goose	2015	5	>10	10	8	2	4	>64	5	4	>64	0.5	0.312	≤0.039	4
MYCAV 179	Trachea	Apátfalva	goose	2015	10	>10	10	16	4	4	4	0.312	4	4	0.5	1.25	≤0.039	8
MYCAV 180	Phallus lymph	Kisbér	goose	2015	5	>10	10	>64	4	4	4	0.312	32	>64	1	1.25	≤0.039	8
MYCAV 202	Cloaca	Kelebia	goose	2015	5	>10	5	16	4	4	32	2.5	0.5	0.5	≤0.25	1.25	≤0.039	8

The following antimicrobial agents were examined during the microbroth dilution tests: the fluoroquinolones: enrofloxacin (batch SZBA336XV), difloxacin (SZBD178XV) and norfloxacin (batch SZBD099XV); the aminoglycoside: spectinomycin (batch SZBB166XV); the lincosamide: lincomycin (batch SZBC340XV); the tetracyclines: doxycycline (batch SZBD007XV) and oxytetracycline (batch SZBC320XV); the macrolides: tilmicosin (batch SZBC345XV) and tylosin (batch SZBB160XV); the pleuromutilins: tiamulin (batch SZBC026XV) and valnemulin (batch SZBE127XV); and the phenicol: florfenicol (batch SZBC223XV); all products originated from VETRANAL, Sigma-Aldrich, Germany. The macrolide tylvalosin (Aivlosin, ECO Animal Health Ltd., UK; LOT M102A) was also included in the examinations. Lincomycin and spectinomycin were applied also in combination at a ratio of 1:2. The antibiotics were diluted and stored according to the recommendations of Hannan [9]. Stock solutions of 1 mg/ml fluoroquinolones were prepared in 0.1 M NaOH; stock solution of 1 mg/ml florfenicol was prepared in 96 % ethanol and in sterile distilled water; and the rest of the stock solutions of 1 mg/ml were prepared in sterile distilled water. Dilutions of the antibiotics were freshly prepared for each microtest from the aliquots stored at −70 °C. Twofold dilutions were prepared in the range 0.039–10 μg/ml for fluoroquinolones, doxycycline and pleuromutilins, 0.25–64 μg/ml for spectinomycin, lincomycin, lincomycin-spectinomycin (1:2) combination, oxytetracycline and macrolides and 0.125–32 μg/ml for florfenicol.

The microbroth dilution examinations on 10^4^–10^5^ CCU/ml of the strains were performed according to Hannan [9]. *Mycoplasma* broth medium was used in the tests as well, and each 96-well microtiter plates contained growth controls (broth medium without antibiotic), sterility controls (broth medium without antibiotic and *Mycoplasma* inoculum) and pH controls (broth medium adjusted to pH 6.8). One clinical isolate (strain MYCAV 65) was selected to be used as quality control of minimal inhibitory concentration (MIC) determination throughout the experiments. The duplicates of three clinical isolates and the duplicate of the selected strain (MYCAV 65) were tested on each 96-well microtiter plates.

The MIC values were determined from the lowest concentration of the antibiotics where no pH and colour change of the broth was detected after one week of incubation, meaning that the growth of the bacteria was completely inhibited in the broth. MIC_50_ and MIC_90_ values were defined as the lowest concentrations that inhibited the growth of 50 % or 90 % of the strains [9].

## Results

The quality control strain (MYCAV 65) showed consistent results throughout the study. Strains with elevated MIC values were found in the cases of all tested antibiotics (Tables 1 and 2).

**Table 2:** Summary of MIC range, MIC_50_ and MIC_90_ values of the isolated *Mycoplasma* sp. 1220 strains

Antibiotic class	Antibiotic agent	Range	MIC_50_	MIC_90_
Fluoroquinolones	Enrofloxacin	1.25 to >10	5	>10
	Norfloxacin	10 to >10	>10	>10
	Difloxacin	1.25 to >10	10	>10
Aminoglycoside	Spectinomycin	4 to >64	8	32
Lincosamide	Lincomycin	2 to >64	4	8
	Lincomycin-spectinomycin (1:2) combination	2 to 32	4	4
Tetracyclines	Oxytetracycline	2 to >64	64	>64
	Doxycycline	0.078 to >10	5	>10
Macrolides	Tylosin	≤0.25 to >64	8	>64
	Tilmicosin	≤0.25 to >64	>64	>64
	Tylvalosin	≤0.25 to 16	0.5	4
Pleuromutilins	Tiamulin	0.156 to 5	0.625	1.25
	Valnemulin	≤0.039 to 0.312	≤0.039	0.078
Phenicol	Florfenicol	2 to 32	8	8

**Fig. 2:**
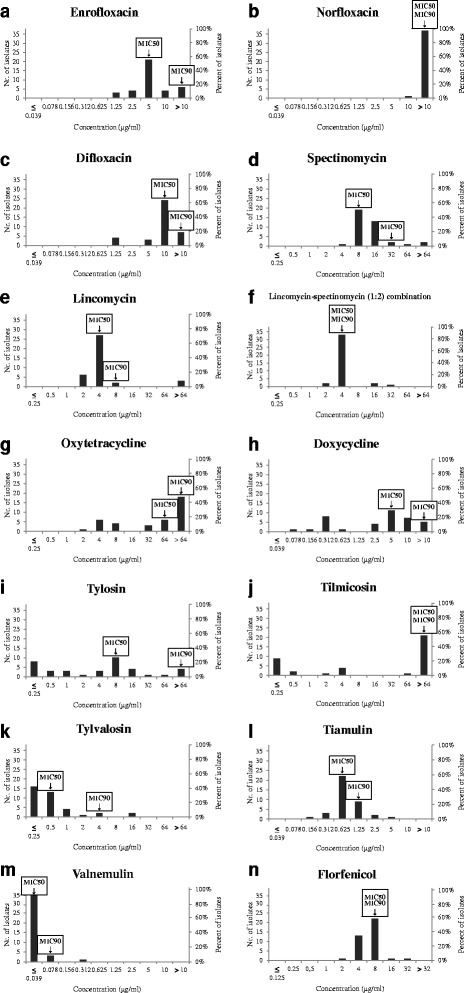
MIC distribution of test antibiotics against *Mycoplasma* sp. 1220 isolates

Among the fluoroquinolones, the MIC values of enrofloxacin and difloxacin showed a wide range (1.25 to >10 μg/ml), while all strains had very high MIC values for norfloxacin (≥10 μg/ml) (Fig. 2a, b and c). The MIC_50_ was 8 μg/ml for spectinomycin and most of the strains yielded the MIC_50_ or higher MIC values (Fig. 2d). The MICs for lincomycin clustered around the MIC_50_ value (4 μg/ml) as well, but high MIC values (>64 μg/ml) were yielded in the case of three isolates (Fig. 2e). The MIC_50_ and the MIC_90_ values (4 μg/ml) for lincomycin-spectinomycin (1:2) combination was the same as the MIC_50_ value for lincomycin. In the case of lincomycin-spectinomycin (1:2) combination the highest concentration needed for inhibition was 32 μg/ml (Fig. 2f). Broad ranges of the MIC values were observed for tetracyclines (2 to >64 μg/ml for oxytetracycline and 0.078 to >10 μg/ml for doxycycline) with high MIC_50_ and MIC_90_ values (Fig. 2g and h). The broadest ranges of MIC values were detected for tylosin and tilmicosin (≤0.25 to >64 μg/ml) with high MIC_50_ and MIC_90_ values in the case of tilmicosin (Fig. 2i and j). While the MIC values for tylosin showed diverse distribution, the strains’ susceptibility profiles formed three groups in the case of tilmicosin (≤0.25, 4 and >64 μg/ml) (Fig. 2j). Among the examined three macrolides (tylosin, tilmicosin and tylvalosin), tylvalosin showed the lowest MIC_50_ value (0.5 μg/ml) against the strains (Fig. 2k). From the pleuromutilins the MIC values of tiamulin were higher than those of valnemulin, and the latter compound was found to be the most active antibiotic in the examinations (Fig. 2l and m). In the case of florfenicol, the susceptibility profiles of most strains were similar to each other and showed the MIC_50_ and MIC_90_ value (8 μg/ml) or its two-fold lower dilution (4 μg/ml) with few exceptions (Fig. 2n).

*M*. sp. 1220 strains isolated year by year from the same farms and from the same tissue types (e.g. strains MYCAV 34, 53 and 67 from Szentes, strains MYCAV 50, 70, 176 and 177 from Cered, or strains MYCAV 38 and 202 from Kelebia) showed elevated MIC values from year to year in the cases of certain antibiotics. Higher MIC values were detected in subsequent isolates for lincomycin, lincomycin-spectinomycin combination, tetracyclines (both oxytetracycline and doxycycline), macrolides (tylosin, tilmicosin and tylvalosin), tiamulin and for florfenicol as well.

## Discussion

Information about the susceptibility of *M*. sp. 1220 strains to antimicrobials is scarce, as until to date the sole published reference concerning the antibiotic susceptibility profile of this species is a review of Stipkovits and Szathmary [3]. Stipkovits and Szathmary determined the values of enrofloxacin, tylosin, chlortetracycline, oxytetracycline, doxycycline, tiamulin and lincomycin in *Mycoplasma* species affecting waterfowl (*M. anatis*, *M. cloacale*, *M. anseris* and *M*. sp. 1220), although detailed data of their method is lacking [3]. Thus we are facing the absence of reports about the antibiotic susceptibility of *M*. sp. 1220 and also of other *Mycoplasma* species occurring in waterfowl. Therefore, the results of the current study are also compared to data of antibiotic susceptibility of the well-studied *Mycoplasma* species of poultry: *M. synoviae* and *M. gallisepticum*.

Elevated MIC values were reported previously in the case of the fluoroquinolones, especially of enrofloxacin in *M*. sp. 1220 (MIC_50_ 2 μg/ml and MIC_90_ 4 μg/ml) and other *Mycoplasma* species of poultry [3, 13, 20, 21]. In addition, the increasing occurrence of quinolone-resistant *M. synoviae* and *M. gallisepticum* field isolates were also observed [13, 22]. In the current study, the detected MIC_50_ values (5 μg/ml for enrofloxacin, 10 μg/ml for difloxacin and ≥10 μg/ml for norfloxacin) were even higher than the ones reported before [3, 13, 20–22], confirming the observation of increasing quinolone-resistance in *Mycoplasma* species. In order to save these antibiotics for human disease treatment the directive was to reduce the use of these agents in livestock. Former efforts for the prevention of the appearance of quinolone-resistant species are proved to be unsuccessful considering the observed dramatic elevations in the MIC values of these antibiotics in avian *Mycoplasma* species [13, 21, 23].

Administration of the combination of lincomycin and spectinomycin could reduce the egg infertility rates and increase the hatching rates and the egg production in *M*. sp. 1220 infected geese [11]. The lincomycin-spectinomycin therapy was proved to be effective against other *Mycoplasma* species as well; however, application of spectinomycin in monotherapy is not recommended for its insufficient effectiveness and relatively high MIC values in in vitro experiments [12]. In vitro effectiveness of lincomycin at 2 μg/ml MIC_50_ values against *M*. sp. 1220, *M. anseris* and *M. anatis* species has been reported [3]. In the present study, all isolates showed elevated MIC values for spectinomycin, lincomycin and lincomycin-spectinomycin combination. The growth of a couple of strains was not inhibited even at the highest concentrations used (64 μg/ml) for spectinomycin and lincomycin individually. The combination of the two antibiotics improved their effectiveness, as lincomycin-spectinomycin combination could inhibit the growth of all examined strains within the concentration range used (0.25 to 64 μg/ml) and lower MIC_90_ value was observed also.

Previously, tetracyclines (chlortetracycline, doxycycline and oxytetracycline) showed 1–2 μg/ml MIC values against *M*. sp. 1220 strains. Growth of other *Mycoplasma* species isolated from waterfowl were inhibited at 2–4 μg/ml MIC_50_ values using the same antibiotics [3]. Previously *Mycoplasma* species infecting poultry were observed to be inhibited by elevated MIC values, although with exceptions, as *M. synoviae* strains showed high susceptibility to doxycycline in the Netherlands [12–14]. In the current study, although the *M*. sp. 1220 strains showed broad ranges of MIC values for oxytetracycline and doxycycline, more than 50 % of the strains were inhibited by only higher antibiotic concentrations (64 and 5 μg/ml, respectively) and MIC_90_ values exceeded the concentration ranges used for both compounds. These results show a dramatic increase of the MIC values of tetracyclines against *M*. sp. 1220 strains and reveals the presence of probably highly resistant strains in Hungary.

Macrolides, especially tylvalosin have good in vitro effectiveness against most *Mycoplasma* species infecting poultry, showing lower MIC values in previous examinations than quinolones and tetracyclines [3, 12–15]. However, *M. gallisepticum* could develop resistance rapidly to these compounds, especially to tilmicosin [24]. Earlier, the MIC_50_ values in *M.* sp. 1220, *M. anatis, M. anseris* and *M. cloacale* strains were defined to be 2 μg/ml for tylosin [3]. In the current study, the MIC_50_ value (8 μg/ml) of tylosin was higher than the previous observation [3], and the MIC_90_ value exceeded the concentration range used in the experiment. However, high variability was observed in the susceptibility of the strains to this compound. Similarly, wide range of the MIC values was detected for tilmicosin, highlighting the necessity of susceptibility testing before antibiotic treatments. As opposed to the diverse susceptibility profiles of the strains for tylosin, the MIC values of tilmicosin were categorized into three separate groups. The observed distribution of the MIC values is likely in association with the capability of *Mycoplasma* sp. 1220 to develop resistance more rapidly to tilmicosin (i.e. with one or two mutations) than to other macrolides. The same phenomenon was described in other *Mycoplasma* species as well [24]. Out of the three macrolides examined in the study, tylvalosin proved to be the most effective agent against *M*. sp. 1220 strains, showing lower MIC_50_ value (0.5 μg/ml) against the pathogen than the majority of the antibiotics tested.

Pleuromutilins showed good in vitro effectiveness against avian *Mycoplasma* species in previous studies and low tendency of the development of resistance to these agents has been reported [16–18, 21]. Tiamulin was used successfully for the treatment of mycoplasmosis and its effectiveness was similar to spectinomycin therapy in the treated geese [11]. Stipkovits and Szathmary described low MIC values (MIC_50_: 0.06 μg/ml, MIC_90_: 0.25 μg/ml) of tiamulin in the case of *M.* sp. 1220, and similarly low MIC_50_ values (0.125–1 μg/ml) were observed against *M. anseris*, *M. anatis* and *M. cloacale* [3]. In the present study, pleuromutilins were found to be the most effective antibiotic agents and the examined compounds, especially valnemulin showed high in vitro effectiveness against all tested isolates of the pathogen. However, it is noteworthy, that strains with elevated MIC values were detected for tiamulin (MIC: 2.5–5 μg/ml) and even for valnemulin (MIC: 0.312 μg/ml). Although the low MIC values of valnemulin against *M*. sp. 1220 strains in vitro are promising for its clinical use, it should be noted that in a previous study only a single mutation in *M. gallisepticum* could cause elevation in the MIC values of valnemulin [17]. To date, the use of pleuromutilins in humans is limited, as only one commercially available product is authorized containing this active substance. However, bacterial strains resistant to pleuromutilins have already been described and these strains also show multidrug resistance, which warrants the prudent use of these antibiotic agents [25].

Phenicols showed good in vitro activity against *Mycoplasma* species of poultry, but information about their effectiveness in waterfowl is lacking [26, 27]. In the present study, most of the *M*. sp. 1220 isolates yielded the same MIC values (4 or 8 μg/ml) for florfenicol, and only two isolates (originating from the same region) showed elevated MIC values compared to the MIC_50_ (8 μg/ml), one of them reaching the highest antibiotic concentration (32 μg/ml) used.

The elevated MIC values of several antibiotics detected in subsequent isolates from the same farms from year to year are likely in association with the inconsistent use of antibiotics, the rapid development of antibiotic resistance and highlight the importance of susceptibility testing before therapy and responsible use of antimicrobial drugs.

## Conclusion

In the present examinations the antibiotic susceptibility profiles of thirty-eight *M*. sp. 1220 strains isolated in Hungary were determined. To the best of our knowledge, this is the first detailed study about the antibiotic susceptibility of *M*. sp. 1220, a pathogen which could cause significant economic losses in waterfowl flocks. Valnemulin, tiamulin and tylvalosin were found to be the most effective antibiotics in the present study. Most of the isolates showed elevated MIC values for more than one agent, but none of the strains yielded high MIC values for all the examined antibiotics. Nevertheless, our results confirmed that increasing resistance could be observed in the cases of several antibiotics. These findings highlight the consistent use of antibiotics and the need for determination of antibiotic susceptibility of *Mycoplasma* species before treatment.

## Abbreviations

MIC: Minimal inhibitory concentrations
